# Assessment of Hepatic and Pancreatic Iron Overload in Pediatric Beta-Thalassemic Major Patients by T2* Weighted Gradient Echo Magnetic Resonance Imaging

**DOI:** 10.1155/2013/496985

**Published:** 2013-03-28

**Authors:** Doaa Mohammed Youssef, Faten Fawzy Mohammad, Ayman Ahmed Fathy, Maha Aly Abdelbasset

**Affiliations:** ^1^Pediatric Department, Faculty of Medicine, Zagazig University, Zagazig, Egypt; ^2^Radiodiagnosis Department, Faculty of Medicine, Zagazig University, Zagazig, Egypt

## Abstract

*Background*. MRI has emerged for the noninvasive assessment of iron overload in various tissues. The aim of this paper is to evaluate hepatic and pancreatic iron overload by T2^∗^ weighted gradient echo MRI in young beta-thalassemia major patients and to correlate it with glucose disturbance and postsplenectomy status. *Subjects and Methods*. 50 thalassemic patients, in addition to 15 healthy controls. All patients underwent clinical assessment and laboratory investigations. Out of 50 thalassemic patients, 37 patients were splenectomized. MRI was performed for all subjects. *Results*. All patients showed significant reduction in the signal intensity of the liver and the pancreas on T2^∗^GRD compared to controls, thalassemic patients who had abnormal glucose tolerance; diabetic and impaired glucose tolerance patients displayed a higher degree of pancreatic and hepatic siderosis and more T2^∗^ drop in their signal intensity than those with normal blood sugar level. Splenectomized thalassemic patients had significantly lower signal intensity of the liver and pancreas compared to nonsplenectomized patients. *Conclusion*. T2^∗^ gradient echo MRI is noninvasive highly sensitive method in assessing hepatic and pancreatic iron overload in thalassemic patients, more evident in patients with abnormal glucose tolerance, and is accelerated in thalassemic splenectomized patients.

## 1. Introduction

*β*-Thalassemia major is a hereditary hemolytic anemia that is treated with multiple blood transfusions. A major complication of this treatment is iron overload, which leads to cell death and organ dysfunction [1]. Iron accumulates initially in the reticuloendothelial system (bone marrow, spleen, and liver) and then in the hepatocytes, the heart (myocytes), and the endocrine glands [2]. The turnover of iron in the hepatocytes, myocytes, and endocrine glands is very low. Chelation therapy has been used to eliminate it [3], methods to estimate total body iron stores are required, and serum ferritin acts as a reliable marker but can yield false results in the presence of inflammation and liver disease [4]. 

Liver is the primary site for iron storage in patients with hemochromatosis or transfusion-dependent anemia; therefore, liver iron concentration (LIC) accurately reflects total body iron stores [5]. Classically, liver iron assessment has been performed by needle biopsy. This procedure carries a 0.5% complication risk and it is also disliked by patients [6].

Clinical management of these patients is also based on the assessment of liver iron stores for several reasons. First, liver iron was shown to correlate closely with total body iron. Second, liver iron concentration is a proven prognostic indicator in thalassemic patients [7].

Impairment of the endocrine and exocrine functions of the pancreas is a common complication in patients with beta-thalassemia major [8]. The incidence of impaired glucose tolerance and diabetes in thalassemia major patients varied from 9% to 15% depending on the age of assessment, the intensity of chelation, transfusion, and related patient compliance [9]. The etiology of diabetes in *β*-thalassemia is increased peripheral resistance to insulin and direct toxic effect of excess iron in the acinar and beta cells of the pancreas resulting in insulin deficiency [10].

Magnetic resonance imaging (MRI) represents the most available noninvasive technique to assess hepatic iron content and shows a good correlation with biopsy results [11, 12]. It is the best noninvasive method for measuring the level of iron in the liver for the purposes of confirming the diagnosis, determining the severity and monitoring therapy with high sensitivity, specificity, and positive and negative predictive values [13].

## 2. Patients and Methods

The study was conducted at the Radiology and Pediatrics Departments, Zagazig University, in the time frame of December 2010 to December 2011, and included 50 thalassemia major pediatric patients (33 males and 17 females), whose age ranged between 9 and 16 years, referred from the Pediatric Hematologic Clinic, in addition to 15 healthy controls. All patients were diagnosed as thalassemia major patients based on clinical and hematological studies. They were on regular blood transfusion 12 times/year and received desferrioxamine as chelation therapy 4 days per week.

The patients were subdivided clinically into three groups as follows.

- *Group A*: included patients with normal glucose level (26/50).
- *Group B*: included patients with impaired glucose tolerance (14/50).
- *Group C*: included thalassemic diabetic patients (10/50).

Splenectomized patients were 37/50 of all our patients. All patients underwent MRI with 1.5 Tesla scanner (Philips Medical System). T1 and T2WIs were obtained in the axial planes plus breath hold gradient echo sequence using a body coil to avoid signal drop-off and to ensure the highest uniformity in the signal-to-noise ratio throughout the scanned volume. 

 The used T2* gradient echo pulse sequence in the axial plane was obtained with 25 slices to cover the whole abdomen using the following:

- TE = 5–10 m/sec TR = 50–120 m/sec,
- flip angle = 20,
- FOV = 350–400 mm,
- matrix (frequency × phase) 256 × 256 pixels,
- slice thickness = 5–7 mm with 1 mm interval,
- in-phase and out-phase MR imaging was also performed in five cases, using TE 4.2 m/sec and 2.1 m/sec, respectively, to differentiate between siderosis and steatosis.

The results were tabulated with statistical analysis of (SPSS version 19) data expressed as mean ± SD or number and percentage. 

## 3. Results

We compared the clinically classified groups of our cases as regards the mean age at diagnosis with laboratory assessment of their serum ferritin level which was significantly higher in patients with abnormal glucose tolerance (groups B and C) compared to nondiabetic patients (group A) (*P* < 0.05) (Table 1).

**Figure 1:**
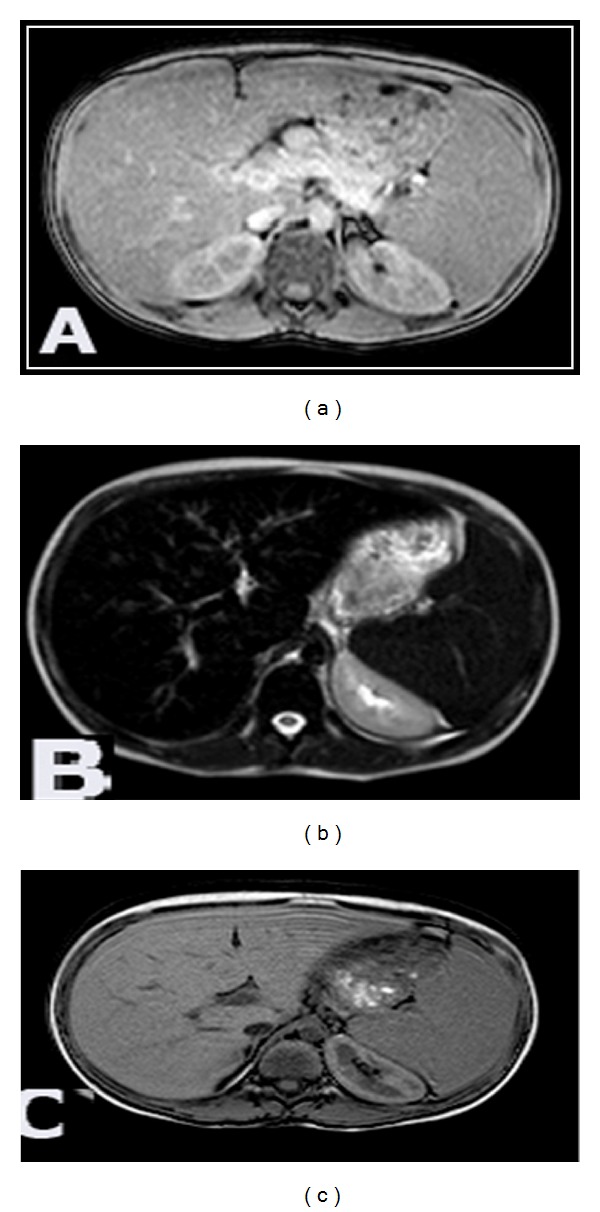
11-year-old thalassemic male patient with glucose intolerance. (a) Axial T2WI showing normal signal intensity of the liver and spleen. (b) T2*GRE axial images revealed drop in the signal intensity of the liver and spleen relative to the paraspinal muscles. (c) Out-phase axial images revealed no signal intensity changes excluding steatosis and confirming siderosis.

 Tissue iron overload was detected in the different groups of thalassemic patients on the basis of decreased signal intensity of the involved organ parenchyma on T2*GRE sequence as a result of the paramagnetic susceptibility of iron and was compared to the signal intensity on T2WI of the same patient and relative to the studied control cases (Figure 1).

**Figure 2:**
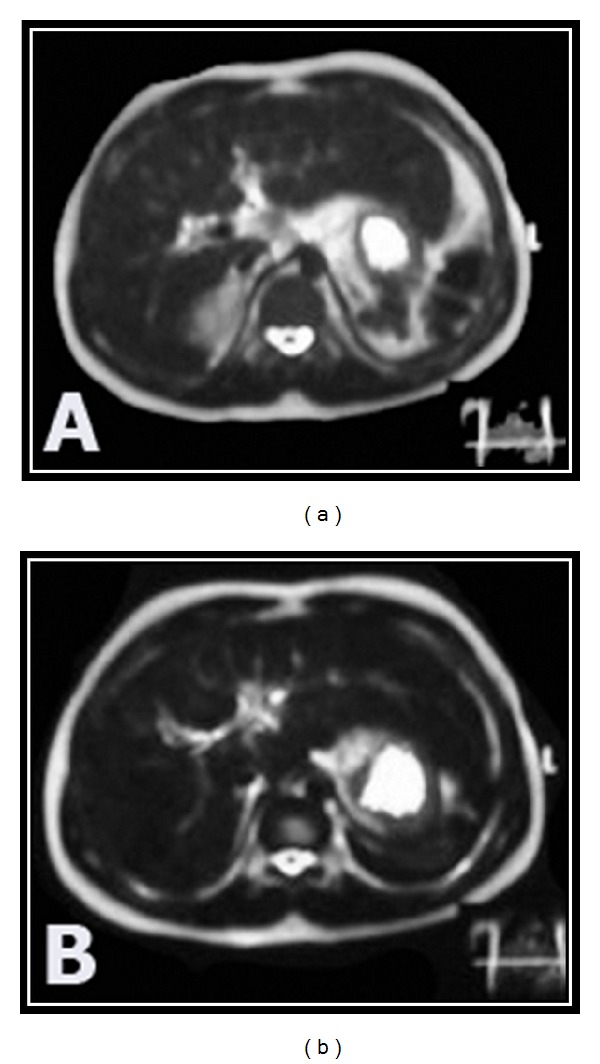
15-year-old thalassemic diabetic female patient. (a) Axial T2WI revealed decreased signal intensity of the liver. (b) Axial T2*GRE images revealed more drop in the signal intensity of the liver.

Hepatic siderosis was diagnosed in 45/50 (90%) of thalassemic patients and pancreatic siderosis in 21/50 of patients (42%). Thalassemic patients with impaired glucose tolerance (group B) and who are diabetic (group C) showed significant reduction in the signal intensity of the liver and the pancreas compared to those with normal glucose tolerance (group A) (Table 2) (Figure 2). 

Patients with impaired glucose tolerance (14 patients) showed normal signal intensity of the pancreatic parenchyma in 10/14 patients and hypointense signal of the pancreatic parenchyma in 4/14 patients while all thalassemic diabetic patients showed significant drop in the pancreatic parenchyma signal intensity (Table 2).

On T2WI, the hepatic siderosis was detected in 28/50 (56%) patients by the hypointensity of the hepatic parenchyma relative to the healthy control hepatic parenchymal signal (11 cases from group A, 17 cases from groups B and C) while pancreatic siderosis was detected in 9 out of 50 patients (18%), 4 cases were with impaired glucose tolerance and 5 cases were diabetic. These findings confirmed that T2*GRE sequence is highly specific and more sensitive to the magnetic susceptibility of the deposited iron than seen on cT2WIs as hepatic siderosis was diagnosed in 90% of cases and pancreatic siderosis in 42% of cases by T2*GRE images compared to 56% and 18%, respectively, by cT2WI.

The drop of the signal intensity of both hepatic and pancreatic parenchymas occurred in both diabetic and impaired glucose tolerance patient groups with no statistically significant difference (*P* > 0.05). 

**Figure 3:**
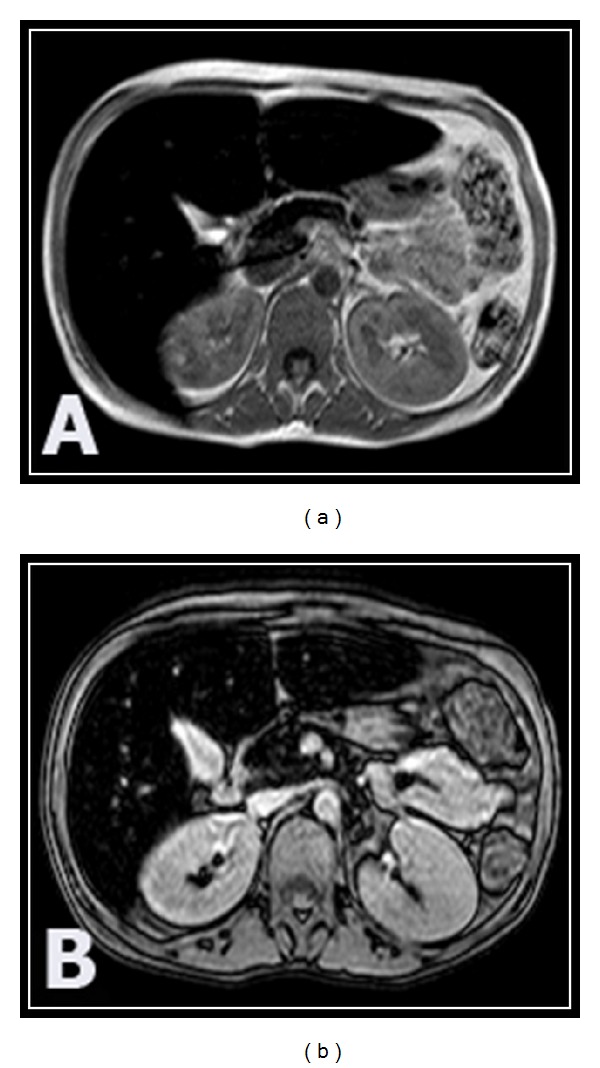
14-year-old splenectomized thalassemic male patient with diabetes. (a) T2*GRE axial images show accentuated decrease in the signal intensity of the liver and pancreas. (b) In-phase axial images show reduction of the signal intensity of the liver and pancreas confirming iron overload and excluding steatosis.

The drop of signal intensity of the liver and pancreas on T2*GRE was accentuated in splenectomized thalassemic patients as seen in 35/37 and 21/37 patients, respectively, compared with those with preserved spleen, particularly in patients with abnormal glucose tolerance (Table 3) (Figure 3).

In- and out-phases MR sequences were used in 5/50 patients to exclude suspected fatty parenchymal infiltration that results in drop of normal SI on the out-phase sequence and we did not detect any case of steatosis in our study (Figures 1 and 3).

## 4. Discussion

Magnetic resonance (MR) imaging is the most sensitive and specific imaging modality in the diagnosis of parenchymal iron overload in thalassemic patients on regular blood transfusion. The susceptibility effect caused by the accumulation of iron leads to signal loss in the affected tissues, particularly with the T2* weighted sequences, which makes the diagnosis of iron overload possible in a noninvasive way, thereby avoiding repeated biopsies [14]. 

The accumulation of iron ions in the tissues, because of the superparamagnetic properties of the iron, causes local distortion in the magnetic fields and relaxation of the spins which results in shortening of the longitudinal relaxation time (T1) and the transverse relaxation time (T2), and particularly the transverse relaxation time as affected by magnetic field inhomogeneity (T2*). This effect causes a loss of signal intensity in the affected organs that is proportional to the iron deposition [15].

Dual sequences MR imaging (gradient in and out phase) demonstrates decreased signal intensity in the affected tissues on the in-phase images compared with the out-phase images. This occurs because the echo time of the in-phase sequence is usually higher than that of the out-phase sequence; therefore, the in-phase pulse sequence is more sensitive to iron deposits because of the increased T2* effect [15].

In the current study, the single breath T2* gradient echo sequence was preferred for its short scanning time making it more convenient in young age and the resulting signal intensity was compared to that on T2WI. Dual phase imaging including in- and out-phase gradient MR imaging was used in five cases to differentiate tissue iron overload from suspected steatosis which demonstrates signal intensity drop in the out-phase sequence and we did not detect any cases of hepatic steatosis in our study. 

In the present study, there is significantly lower signal intensity of the liver (45/50) and pancreas (21/50) in thalassemic patients compared to controls on T2*GRE images and in the in-phase sequence in consistency with Matter et al., 2010 [16]. The pancreatic siderosis that resulted in drop of MR signal intensity subsequent to iron overload occurred in 42% of our patients whereas higher percentage was reported by Noetzli et al., 2009 [17], who reported that iron overload in the pancreas occurs in up to 75–100% of thalassemic major cases. This could be explained by difference in the sample size included in the study.

T2*GRE sequence was found to be sensitive to parenchymal iron overload and more specific than conventional T2WI which is diagnosed only in 28/50 (56%) hepatic siderosis cases and 9/50 (18%) pancreatic siderosis cases compared to 45/50 (90%) and 21/50 (42%) cases detected by T2*GRE sequence, respectively. 

Our study also detected lower signal intensity of the liver and pancreas in thalassemic patients with abnormal glucose tolerance (including groups B and C) compared to patients with normal glucose tolerance (group A) in agreement with Matter et al., 2010 [16]. 

In our study, no significant difference between diabetic and impaired glucose tolerance patients in the dropped signal intensity of the liver and pancreas on T2*GRE was detected in the study performed by Mong et al., 2001 [18], who explained the presence of other causative factors for diabetes as genetic predisposition and immune damage.

The reduction in the signal intensity of the liver in thalassemic patients with abnormal glucose tolerance (groups B and C) compared to nondiabetic patients (group A) was in agreement with Papakonstantinou et al., 2007 [19]. This is an important point that reflects the importance of hepatic iron deposition in the development of insulin resistance in consistency with Chern et al., 2001, [20].

Thirty seven patients undergone splenectomy, confirmed by ultrasonography, and all of them showed significant reduction in the signal intensity of the liver and pancreas compared to those with intact spleen. This finding was explained by the study made by Matter et al., 2010 [16], by decreased extrahepatic iron buffering capacity in splenectomized patients with accelerated iron deposition in the liver and pancreas.

## 5. Conclusion

 Hepatic and pancreatic siderosis is a major problem in thalassemic patients and could be detected noninvasively by the drop of signal intensity on T2* weighted gradient echo MRI in the in-phase sequence. It was more evident in patients with abnormal glucose tolerance and is accelerated after splenectomy especially in the pancreas.

## 6. Recommendation

 We recommend T2*GRE sequence in the protocol of MRI regular followup of thalassemic patients and for those under intensive chelation regimen as noninvasive tool to assess improvement of the hepatic and pancreatic siderosis.

## Figures and Tables

**Table 1:** Clinical and laboratory data of the studied groups.

Parameters	Group A (*n* = 26)	Group B (*n* = 14)	Group C (*n* = 10 )	*P*
Age/years	9.8 ± 2.1	10 ± 1.4	11 ± 1.3	0.23
Age at diagnosis	1.4 ± 0.8	1.3 ± 0.9	1.5 ± 0.5	0.82
Serum ferritin level ng/ml	1432 (509) (711–3000)	1765 (765) (1123–4200)	2018 (400) (1000–4678)	0.014*

*Significant*.*

**Table 2:** MR signal intensity of the liver and pancreas in the three studied groups on T2*GRE.

Signal intensity	Group A *N* = 26	Group B *N* = 14	Group C *N* = 10	*P*
Liver				
Normal SI	4	1	0	0.3
Low SI	18	12	1	0.001*
Dark (lower) SI	4	1	9	0.001*
Pancreas				
Normal SI	19	10	0	0.001*
Low SI	7	4	10	0.001*

*Significant*.*

**Table 3:** T2*GRD low SI in splenectomized thalassemic patients (37/50).

Signal intensity	Group A *N* = 18	Group B *N* = 10	Group C *N* = 9	*P*
Liver				
Normal	1	0	1	0.5
Relative low	14	8	6	0.76
Lower SI	3	2	2	0.9
Pancreas				
Normal	31	2	1	0.86
Relative low	3	7	5	0.67
Lower SI	2	1	3	0.27
